# Posterior Microphthalmia, Peripheral Pigmentary Retinal Changes, Yellow Lesions, and Cleft Lip: A Case Report and Literature Review

**DOI:** 10.1155/2019/8392329

**Published:** 2019-05-19

**Authors:** Nasser G. Alsaedi, Khalid Alrubaie

**Affiliations:** ^1^King Khaled Eye Specialist Hospital, Riyadh, Saudi Arabia; ^2^King Abdullah Medical City, Makkah, Saudi Arabia

## Abstract

**Purpose:**

Posterior microphthalmia is a sporadic or inherited developmental ocular anomaly that may occur isolated or in association with multiple ocular and systemic anomalies. This report documents a case of posterior microphthalmia with atypical presentation including white dots in the posterior pole in addition to systemic anomalies including facial defect that can represent an underlying genetic mutation.

**Method:**

Case report.

**Results:**

A 29-year-old male with high hyperopia and history of bilateral clear lens presented with pigmentary changes and white-yellow dots in the posterior pole in both eyes. Patient had a history of cleft lip repair. A complete ocular evaluation including A/B scan and optical coherence tomography confirmed the diagnosis of posterior microphthalmia with a retinitis pigmentosa like fundus and drusen deposits in the subretinal pigment epithelium.

**Conclusion:**

The white-yellow drusenoid deposits in the posterior pole in association with posterior microphthalmia are poorly documented in the literature. Cases of craniofacial developmental defects in association with posterior microphthalmia may represent a genetic defect.

## 1. Introduction

Posterior microphthalmia is a sporadic or inherited developmental ocular anomaly that may occur isolated or in association with multiple ocular and systemic anomalies. Ocular association including retinitis pigmentosa and optic disc drusen were first published by Buys et al. in 1999 [1]. In 2017, Plaza et al. documented white dots in the posterior pole as a new ocular finding associated with posterior microphthalmia [2]. Microphthalmia has been reported to be associated with multiple systemic anomalies including, mental retardation, craniofacial malformations (e.g., cleft lip/palate), and anomalies of the hands and feet and can be a finding in other congenital syndromes [3]. We document a rare case of posterior microphthalmia with other ocular and extraocular anomalies.

## 2. Case Report

A 29-year-old Saudi male presented to our institution (King Khaled Eye Specialist Hospital, KKESH) on 2007 seeking a refractive procedure. The patient had a history of cleft lip repair and had no systemic illness at presentation. The patient denied ocular trauma, ocular surgery or a family history of visual dysfunction. On ocular examination, the visual acuity in the right eye (OD) was 20/50 with a subjective cycloplegic refraction of +15.25 - 0.75 x 140° and 20/30 in the left eye (OS) with a subjective cycloplegic refraction of +15.00 -0.50 x 30°. The interpupillary distance was 63 mm. Intraocular pressure in both eyes (OU) was 19 mmHg. Slit lamp examination was unremarkable OU. White-to-white corneal measurements were 12.2 mm OD and 11.5 mm OS as measured with the slit lamp. The steepest keratometry (K) was 48.3 D at 31° OD and 48.8 D at 116° OS. The corneal thickness measurements were 512 *μ*m OD and 511 *μ*m OS. The biometric measurements as measured by (Orbscan IIz, Bausch and Lomb, Rochester, NY, USA) of the anterior chamber depth revealed 3.32 mm OD and 3.49 mm OS. Iris examination indicated patent peripheral YAG laser iridotomies bilaterally without correctopia OU Piggyback intraocular lenses were present OU.

Indirect ophthalmoscopy was remarkable for crowded optic discs and subretinal drusenoid yellow-white dots symmetrically distributed in the posterior pole OU. Bilaterally, the retinal blood vessels appeared normal, with no clinically obvious papillomacular folds and peripheral pigmentary bone spicule pigmentation.

**Figure 1:**
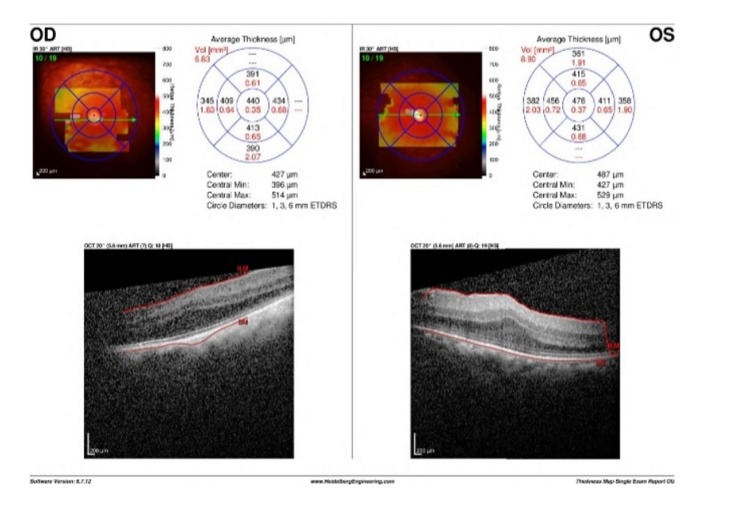
Spectral domain optical coherence tomography of both maculae and horizontal cut, depicting an inverted U-shaped papillomacular folds of the neurosensory retina, greater in the left eye with preservation of the majority of outer retinal layers.

**Figure 2:**
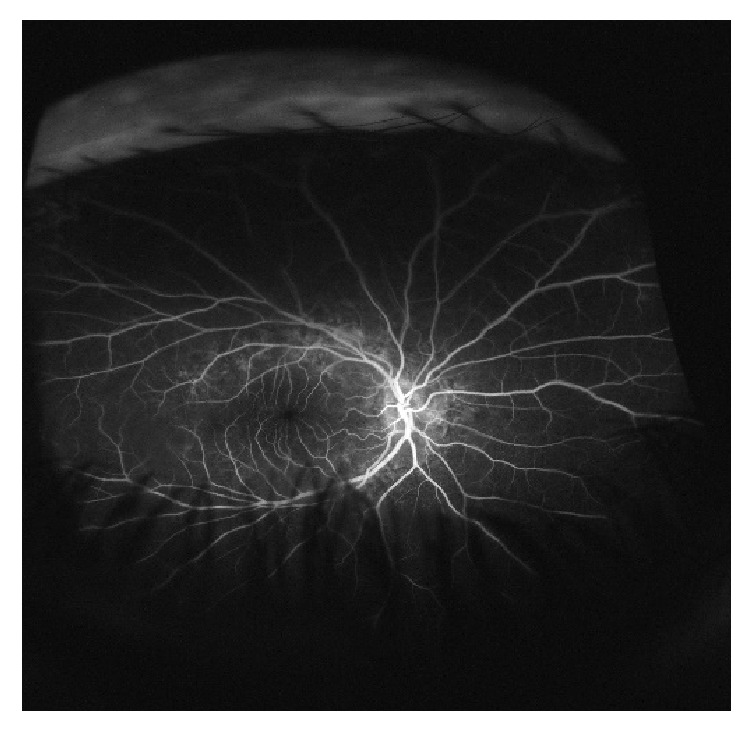
Fundus fluorescein angiography in later phases showing hyperfluorescent deposits in the posterior pole without leakage.

Macular spectral-domain optical coherence tomography (SD-OCT) revealed inverted U-shaped papillomacular folds OU (Figure 1). Posterior microphthalmia was then suspected and A–scan axial length measurements were 16.40 mm OD and 16.65 mm OS. Fluorescein angiography showed no optic nerve head staining or leakage. Staining of the yellowish subretinal drusenoids deposits was seen in the later frames OU (Figure 2). The diagnosis of posterior microphthalmia was confirmed with the given findings and no further intervention was recommended.

## 3. Discussion

Posterior microphthalmia is a rare developmental anomaly and is defined as a normal eye with multiple ocular findings that includes high hyperopia, normal or subnormal anterior segment structures, short axial length that may vary from 12.30 mm to 20.36 mm, papillomacular folds, pseudo-papilledema, thick choroid, and sclera [4]. Posterior microphthalmia is considered a rare subtype of microphthalmia in which there is total axial length reduction with a normal or near normal cornea. Microphthalmos is defined as a structural defect with total axial length at least two standard deviations below age-matched controls or an anteroposterior diameter less than 20 mm in adults [5, 6]. Microphthalmia is categorized generally into pure and complex, pure if there are no associated ocular defects and complex if there are other associated major ocular defects [7]. Our case report presented with ocular and systemic associations.

The association of microphthalmia with pigmentary retinal changes has been previously reported. In 1958, Hermann described 13 patients of 4 generations in a family that showed autosomal dominant transmission of microphthalmia with a variety of retinal pigmentary changes. Hermann also documented two cases reported by Catsch that had microphthalmia with retinitis pigmentosa [8].

In 1965, Franceschetti et al. described a nonconsanguineous family comprised of four individuals with microphthalmia, retinal degeneration, and dental anomalies. They postulated a probable autosomal recessive pattern of inheritance in this association (MIM 251700) [9]. In 1999, Buys et al. published the first combination of nanophthalmos, retinitis pigmentosa, and optic disc drusen that was observed in a 68-year-old male and they proposed that a retinitis pigmentosa-like presentation in such patients may be due to chronic choroidal effusion and serous retinal detachments [1]. These associations described by Buys et al. were subsequently shown to be associated with a frameshift mutation of MFRP (Membrane-type Frizzled-Related Protein) confirming the syndrome of posterior microphthalmos, retinitis pigmentosa, foveoschisis, and optic disc drusen [10]. Interestingly, the association of optic disc drusen with retinitis pigmentosa is well known in the literature. Grover et al. reviewed 262 patients with retinitis pigmentosa and found that 9.2% had optic disc drusen [11]. Another ocular finding in our case is the white/yellow dots in the posterior pole. These white dots in the posterior pole in association with posterior microphthalmia are similar to a case first reported by Plaza et al. in 2017 with posterior microphthalmia, retinitis pigmentosa, optic disc drusen syndrome, and additional findings of yellow macular dots that were not included as criteria for this syndrome [2].

Ayala-Ramires et al. described the criteria for the syndrome as follows: posterior microphthalmos with anteroposterior diameter between 13 mm and 18.5 mm; normal corneal and anterior chamber diameters; high hyperopia, between +8.00 and +25.00 D; optic nerve head drusen with campimetric defects similar to those caused by simple chronic glaucoma; retinal dystrophy compatible with pigment retinosis confirmed by electroretinogram in association with foveoschisis, cystoid macular edema, or foveolar thickening [12]. The yellow dots reported by Plaza et al. were located at the level of retinal pigmented epithelium and the fluorescein angiography indicated a broad window defect with hyperfluorescence of these dots in the later phases [2]. These yellow dots behave like drusen and we postulate that the association of this syndrome with drusen can be found in the disc or the retina.

Papillomacular fold is an ocular finding in posterior microphthalmia and is considered one of the causes of reduced vision. It is confined only to the neurosensory layer sparing the retinal pigment epithelium, choroid, and sclera [13]. It is important for the ophthalmologist to be aware of this finding as this can be confused or mistaken as cystoid macular edema [14]. Additionally, we recommend axial length measurements in patients with hyperopia over +8.00 D and similar posterior segment findings. The SD-OCT finding in our case indicates absence of the normal foveal pit or* fovea plana* that is also associated with microphthalmos, ocular albinism, retinopathy of prematurity, and aniridia [15, 16]. The term fovea plana instead of foveal hypoplasia was suggested by Marmor when the cone is preserved both anatomically and functionally despite the absence of the foveal pit [17]. Fovea plana can be found in normal eyes and up to a 3% incidence had been reported in children with clinically normal eyes [18].

The extraocular manifestation of patients with microphthalmia spectrum includes multisystemic malformations of the heart, renal, face, and central nervous system [19]. A systemic work-up should be performed in patients with multiple malformations and a chromosomal microarray study or exome sequencing may be required in those with multiple malformations (Table 1) [3].

## 4. Conclusion

Posterior microphthalmia should be suspected in cases with high hyperopia. The occurrence of posterior microphthalmia with other ocular and systemic associations has been reported in the literature. The white-yellow dots in the posterior pole in posterior microphthalmia are an atypical finding and poorly described in the literature. Cases of craniofacial developmental defects in association with ocular anomalies including posterior microphthalmia may represent genetic/chromosomal defects. We recommend evaluating such cases to establish a distinct pattern of inheritance.

## Figures and Tables

**Table 1:** A Practical Classification of Microphthalmia/Coloboma.

*Isolated*

Microphthalmia
Colobomatous
Isolated uveoretinal coloboma
Microphthalmia with cyst
Non-colobomatous

*Microphthalmia with Ocular Anomalies*

Cataract
Myopia and corectopia
Ectopia lentis
congenital retinal detachment
Persistent Hyperplastic Primary Vitreous

Aicardi syndrome

*Microphthalmia with Mental Retardation*

Mental retardation
Mental retardation and congenital spastic diplegia (Sjogren-Larsson)

*Microphthalmia with Craniofacial Malformations*

Facio-Auriculo-Vertebral sequence
Hallermann-Streiff syndrome
Amniotic band syndrome
Transverse facial cleft
Cleft lip/palate
Microcephaly
Microcephaly and retinal folds
Hydrocephalus and congenital retinal non-attachment (Warburg syndrome)

*Microphthalmia with Malformations of the Hands and Feet*

Polydactyly
Waardenburg's recessive anophthalmia syndrome

*Microphthalmia with Multiple Congenital Anomalies (Syndromes)*

CHARGE association
Duker syndrome
Lenz microphthalmia syndrome
Oculo-Dento-Osseous Dysplasia
Cryptophthalmos syndrome
Cerebro-Oculo-Facial Syndrome
Goltz syndrome or focal dermal hypoplasia
Lowe syndrome
Meckel-Gruber syndrome
Basal cell nevus syndrome of Gorlin-Goltz
Cross syndrome
Microphthalmia with linear skin defects

*Microphthalmia in Chromosomal Anomalies*

T-13 (Patau)
4p- (Wolf-Hirschorn)
18q-
18r
T-18 (Edward)
Cat-eye syndrome (marker 22)
Other chromosomal aberrations

*Microphthalmia and Intrauterine Insults*

Maternal drug intake: thalidomide, alcohol, isotretinoin, others
Maternal vitamin A deficiency
Maternal fever or radiation exposure
Maternal uncontrolled phenylketonuria
Intrauterine infections: CMV, EBV, Varicella, Herpes simplex,
Rubella, Toxoplasmosis

Adopted from [3].
